# Depression in middle-aged and older adults with hearing loss: the use and construction of a nomogram tool

**DOI:** 10.3389/fpubh.2024.1452285

**Published:** 2024-12-11

**Authors:** Qiankun Liu, Zhongtao Zhou, Yang Xu, Jiaxue Pang, Chunlu Zeng, Xiaoqing Ma, Pengyao Li, Ma Li, Juju Huang, Hui Xie

**Affiliations:** College of Nursing, Bengbu Medical University, Bengbu, China

**Keywords:** hearing loss, depressive symptoms, middle-aged and older adult people in community, nomogram, CHARLS

## Abstract

**Objective:**

This study aims to explore the influencing factors of depressive symptoms in middle-aged and older adult patients with hearing loss and to construct a nomogram risk prediction model.

**Methods:**

A total of 2,729 middle-aged and older adult patients with hearing loss from the community were selected as the study subjects. Single-factor and multifactorial stepwise logistic regression analyses were used to screen influencing factors. Based on these predictive factors, a nomogram prediction model was constructed. The model’s efficacy was validated using the area under the receiver operating characteristic curve (AUC) and 1,000 bootstrap resamples.

**Results:**

Multifactorial logistic regression analysis revealed that age, gender, pain, cognitive abilities, daily living abilities, sleep duration, and self-rated health status are the main influencing factors for depressive symptoms. Based on these factors, the constructed nomogram model demonstrated good calibration (χ^2^ = 3.367, *p* = 0.909), with an AUC value of 0.741 (95% CI: 0.723–0.759), and a sensitivity of 0.683, showing good discriminative ability and accuracy.

**Conclusion:**

The incidence of depressive symptoms is relatively high among middle-aged and older adult individuals with hearing loss. The model developed in this study can effectively identify middle-aged and older adult patients with hearing loss who are at risk of depressive symptoms. This provides strong empirical support for the early detection, diagnosis, and intervention of depressive symptoms in middle-aged and older adult patients with hearing loss.

## 1 Introduction

With the accelerated advent of an aging society, hearing loss has become one of the most common sensory impairments affecting the older adult. According to the 2019 Global Burden of Disease data, over 1.5 billion people suffer from the challenges of hearing loss, with approximately 403 million individuals (representing 26%) facing moderate to severe reductions in hearing (1). During the same period, the number of individuals with hearing loss in China reached 407 million, and with the accelerated aging of the population, the demand for rehabilitation among hearing loss patients has significantly increased (2). Research reports from around the world have consistently confirmed that the incidence of this condition is high globally and increases significantly with age (3–5). However, prolonged hearing loss is not merely a physiological issue; it severely impacts an individual’s communication abilities, social and psychological health, and quality of life. This can lead to a range of psychosocial issues, including social isolation, depressive symptoms, and cognitive impairments (6–9).

There is a close connection between depressive symptoms and hearing loss; studies show that the incidence of depressive symptoms is significantly higher among those affected by hearing loss compared to the general population (8). Given that depressive symptoms can be alleviated through pharmacological treatments and psychosocial interventions, prompt diagnosis and treatment are particularly critical. However, diagnosing depressive symptoms in middle-aged and older adult populations affected by hearing loss poses increased challenges, often leading to misdiagnosis or neglect due to communication difficulties and physical symptoms (such as headaches and dizziness) caused by hearing impairment (10). Consequently, depressive symptoms in this group often fails to receive adequate attention, correct diagnosis, and timely treatment. Depressive symptoms is not only one of the major causes of disability and suicide globally, but it is also considered an important predictor of the risk of suicide and physical decline in the older adult (11–13).

Timely diagnosis of depressive symptoms in middle-aged and older adult populations is crucial, yet often delayed or not effectively managed due to various factors. These factors include insufficient awareness of depressive symptoms, the diversity of symptoms, and reduced attention to the psychological state of the older adult by family members and society. Additionally, the influence of different socio-cultural backgrounds and health behaviors on this issue cannot be ignored. In China, traditional cultural views on mental health tend to be conservative, with older adult individuals often choosing to endure their psychological distress, and family members more likely to focus on physical health rather than psychological needs. With the increasing prevalence of hearing loss among middle-aged and older adult populations, although some studies have explored the factors related to depressive symptoms in this group, research that utilizes epidemiological survey data to deeply analyze the relationship between hearing loss and depressive symptoms characteristics remains scarce. Therefore, identifying these factors is essential for the early recognition, prevention, and treatment of depressive symptoms. Previous studies have shown that age, gender, educational level, socio-economic status, lifestyle, and psychological stress can all influence the occurrence of depressive symptoms in the older adult. However, these studies are often limited by sample selection, such as narrow geographical scope and insufficient generalization of study subjects. Currently, there is no specific predictive model for the risk of depressive symptoms among middle-aged and older adult individuals with hearing loss. Given the high prevalence and harm of hearing loss and depressive symptoms in this population, as well as the limitations of current research, this study aims to develop a predictive model for depressive symptoms risk in middle-aged and older adult individuals with hearing loss. It seeks to comprehensively assess the risk factors for depressive symptoms in this group to identify high-risk individuals early and provide a basis for reducing incidence rates and developing personalized intervention measures.

## 2 Methods

### 2.1 Study design and participants

**Figure 1:**
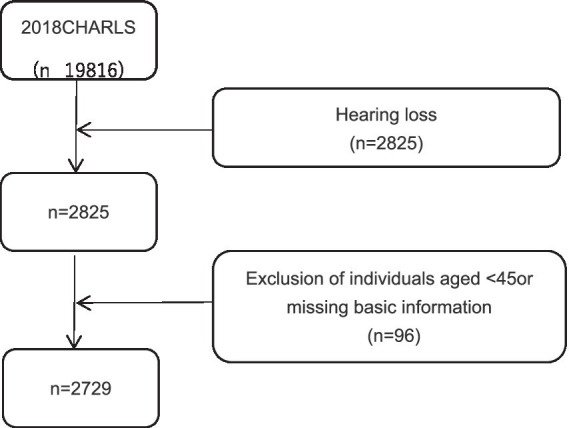
CHARLS selection process flowchart.

Our research subjects were drawn from the China Health and Retirement Longitudinal Study (CHARLS) 2018 survey participants. CHARLS is a national longitudinal survey targeting the Chinese population aged 45 or older and their spouses, covering 29 provinces (including autonomous regions and municipalities), 150 counties, and 450 communities (villages). The baseline study was initiated in 2011, with biennial assessments and follow-ups involving physical measurements. The CHARLS database comprises seven modules, including basic personal information, family information, health status and function, medical care and insurance, employment, retirement and pensions, income, expenditure and assets, and housing conditions, as well as detailed physical examinations and blood tests. Participants who met the criteria were selected from CHARLS 2018 for this study’s analysis. The data complies with the Declaration of Helsinki and was approved by the Institutional Review Board of Peking University (IRB00001052-11015) (14). The inclusion criteria were as follows: (1) aged ≥45 years; (2) self-reported poor hearing based on survey question DA039 (“How would you rate your hearing ability? Excellent, very good, good, fair, or poor”); (3) complete survey data on basic information, depressive symptoms, daily living abilities, sleep, self-assessed health status, hospitalization conditions, use of hearing aids, and chronic conditions. Out of 19,816 individuals aged 45 and above surveyed in CHARLS 2018, 2,825 self-reported hearing loss, and 2,729 participants met the eligibility criteria and were included in the study. As illustrated in Figure 1.

### 2.2 Predictive factors

#### 2.2.1 Assessment of depressive symptoms

In the CHARLS survey, a simplified version of the CES-D10 (Center for Epidemiologic Studies Depressive symptoms Scale) was integrated into the questionnaire (questions DC009-018) to assess the depressive symptoms levels of respondents. The Chinese version of this scale has been applied in the older adult population in China and has shown excellent reliability and validity, with a total Cronbach’s alpha coefficient of 0.815 (15). And this scale has been used in multiple studies involving hearing-impaired populations (16). Each question in the questionnaire is scored on four levels: “Rarely or none of the time” (0 points), “Some or a little of the time” (1 point), “Occasionally or a moderate amount of time” (2 points), and “Most or all of the time” (3 points). The maximum score is 30 points, and a higher score indicates a greater risk of depressive symptoms. In these 10 questions, items 5 and 8 are scored in reverse; the remaining eight items are scored 0 points for “Rarely or none of the time” and increase accordingly. In this study, participants scoring 10 or above on the CES-D10 were categorized as at risk of depressive symptoms, while scores below 10 were considered within the normal range (17).

#### 2.2.2 Demographic characteristics

Demographic characteristics include age, gender (0 = female, 1 = male), marital status (0 = separated/divorced/single/widowed, 1 = married and cohabiting), place of residence (0 = urban, 1 = rural), educational level (0 = illiterate, 1 = less than 6 years of education, 2 = 6 years or more of education), family income (0 = less than 10,000, 1 = 10,000–29,999, 2 = 30,000–49,999, 3 = 50,000 and above), number of children in the family (0 = none, 1 = one, 2 = two, 3 = three, 4 = four or more), and retirement status (0 = no, 1 = yes).

#### 2.2.3 Health status and behaviors

Health status and behaviors include activities of daily living (ADL), sleep duration, and physical pain. The ability to perform daily activities is assessed using the ADL scale, which includes six activities: dressing, bathing, eating, getting in or out of bed, toileting, and controlling urination and defecation. These are categorized as “no difficulty,” “some difficulty but can manage,” “difficulty and needs help,” and “completely unable to perform.” Any difficulty in performing any of these tasks is considered a limitation in ADL functionality (18). Night-time sleep duration is classified into four categories (0 = less than 4 h, 1 = 4–5.9 h, 2 = 6–7.9 h, 3 = 8 h or more). Physical pain is assessed based on the question DA041 “Do you often suffer from pain?,” with responses categorized as either present or absent (0 = no, 1 = yes).

#### 2.2.4 Psychological and mental health factors

This category includes self-assessed health and cognitive abilities. Self-assessed health is measured by the question: “How do you rate your current health status?” Answers range from very good, good, average, poor, to very poor (0 = poor/very poor, 1 = very good/good/average). Cognitive ability is a continuous variable assessed using the MMSE (Mini-Mental State Examination) scale, which evaluates fixed and fluid abilities. This includes assessing situational memory capabilities (such as memory tests and delayed memory tests), mental state (orientation recognition tests, calculation abilities, and drawing ability tests). The scoring range is 0 to 30 points, with higher scores indicating better cognitive function (19).

### 2.3 Statistical analysis

Statistical analyses were performed using SPSS (version 25.0) and R (version 3.5.2). Categorical variables were summarized as frequencies and percentages, while continuous data were represented as mean values, standard deviations, medians, and interquartile ranges. The Chi-square test and Mann–Whitney U test were used for comparisons between groups. Univariate and multivariate logistic regression models were employed to analyze patient clinical data and identify independent risk factors for depressive symptoms. These independent risk factors were incorporated into R software (version 3.5.2), and the RMS package was used to construct a nomogram model to predict the risk of depressive symptoms in patients with hearing loss. ROC curves were plotted, and the area under the curve (AUC) was calculated to evaluate the discriminative ability of the model. Calibration plots and the Hosmer-Lemeshow goodness-of-fit test were used to assess model accuracy. Risk stratification was based on the optimal cutoff value determined by the maximum Youden’s index (BCRL risk probability at maximum Youden’s index, where Youden’s index = sensitivity + (specificity − 1)). Decision curve analysis was utilized to evaluate clinical efficacy. A *p*-value of less than 0.05 was considered statistically significant.

## 3 Results

### 3.1 Characteristics of participants

A total of 2,729 patients participated in this study. The incidence rate of depressive symptoms risk among middle-aged and older adult individuals with hearing loss was 42.4%. Table 1 displays the clinical characteristics of the study sample. The average age of the normal group was 69 (interquartile range: 61–78 years), while the average age of the depressive symptoms group was 65 (interquartile range: 56–72 years). Univariate comparisons between the normal and depressive symptoms groups revealed statistically significant differences (*p* < 0.05) in gender, marital status, area of residence, self-assessed health status, daily living abilities, sleep duration, hospitalization, family income, number of children, number of chronic diseases, use of hearing aids, pain conditions, and hyperlipidemia.

**Table 1:** Characteristics of the study sample (*n* = 2,729).

Factors	Overall (*n* = 2,729)	Non-depression (*n* = 1,571)	Depression (*n =* 1,158)	Statistic	*p*
Age, *M* (Q1, Q3)	67 (59, 76)	69 (61, 78)	65 (56, 72)	111.108a	<0.001
Gender, *n* (%)				47.499b	<0.001
Woman	1,482 (54)	764 (49)	718 (62)		
Man	1,247 (46)	807 (51)	440 (38)		
Education level, *n* (%)				5.668b	0.059
0 year	999 (37)	568 (36)	431 (37)		
<6 years	1,180 (43)	662 (42)	518 (45)		
≥6 years	550 (20)	341 (22)	209 (18)		
Marriage status, *n* (%)				0.899b	0.016
Married	1,949 (71)	1,120 (71)	829 (72)		
Other	780 (29)	451 (29)	329 (28)		
Homeplace, *n* (%)				5.681b	0.017
Urban	1,126 (41)	679 (43)	447 (39)		
Rural	1,603 (59)	892 (57)	711 (61)		
Self-rated health, *n* (%)				193.306b	<0.001
Poor/very poor	1,334 (49)	588 (37)	746 (64)		
Very good/good/average	1,395 (51)	983 (63)	412 (36)		
Activities of daily living, *n* (%)				50.09b	<0.001
Normal	1,776 (65)	1,110 (71)	666 (58)		
Disabled	953 (35)	461 (29)	492 (42)		
Hospitalization status, *n* (%)				9.848b	0.002
None	2,056 (75)	1,219 (78)	837 (72)		
Yes	673 (25)	352 (22)	321 (28)		
Family income, *n* (%)				35.806b	<0.001
<10,000/year	1,310 (48)	713 (45)	597 (52)		
10,000–29,999/year	597 (22)	324 (21)	273 (24)		
30,000–49,999/year	319 (12)	186 (12)	133 (11)		
>50,000/year	503 (18)	348 (22)	155 (13)		
Number of children in the family, *n* (%)				13.459b	0.009
0	53 (2)	35 (2)	18 (2)		
1	272 (10)	162 (10)	110 (9)		
2	785 (29)	422 (27)	363 (31)		
3	707 (26)	392 (25)	315 (27)		
4 or more	912 (33)	560 (36)	352 (30)		
Retirement status, *n* (%)				11.905b	<0.001
Other	1,485 (54)	810 (52)	675 (58)		
Retired	1,244 (46)	761 (48)	483 (42)		
Cognitive score, *M* (Q1, Q3)	7 (1, 15)	5 (0, 15)	9 (4, 14)	740053a	<0.001
Sleep, *n* (%)				123.149b	<0.001
<4 h	459 (17)	178 (11)	281 (24)		
4 ~ 5.9 h	724 (27)	376 (24)	348 (30)		
6 ~ 7.9 h	827 (30)	523 (33)	304 (26)		
≥8 h	719 (26)	494 (31)	225 (19)		
Chronic diseases, *n* (%)				19.214b	<0.001
0	1,264 (46)	777 (49)	487 (42)		
1	823 (30)	466 (30)	357 (31)		
2 or more	642 (24)	328 (21)	314 (27)		
Hearing aid use, *n* (%)				5.336b	0.021
Not uses	2,684 (98)	1,537 (98)	1,147 (99)		
Uses	45 (2)	34 (2)	11 (1)		
Pain status *n* (%)				122.703b	<0.001
Non	701 (26)	529 (34)	172 (15)		
Yes	2,028 (74)	1,042 (66)	986 (85)		
History of alcohol use, *n* (%)				3.472b	0.062
Non	1,795 (66)	1,010 (64)	785 (68)		
Yes	934 (34)	561 (36)	373 (32)		
Hypertension, *n* (%)				2.144b	0.143
Non	2,209 (81)	1,287 (82)	922 (80)		
Yes	520 (19)	284 (18)	236 (20)		
Hyperlipidemia, *n* (%)				15.558b	<0.001
Non	2,377 (87)	1,403 (89)	974 (84)		
Yes	352 (13)	168 (11)	184 (16)		

a: Mann–Whitney U test.

b: Х2 test.

Additionally, a thorough multicollinearity check was conducted on the independent variables (e.g., hearing loss and age) using SPSS (version 23.0) before including them in the regression model. The variance inflation factors (VIF) for all variables were found to be below 5, indicating no collinearity issues. Thus, we can confirm that the independent variables used in the regression analysis do not exhibit multicollinearity.

### 3.2 Logistic regression analysis of influence factors

Following the results of univariate analysis, a multivariate logistic regression was conducted with depressive symptoms occurrence as the dependent variable, incorporating 16 factors that had *p* values less than 0.05 from the univariate analysis. Table 2 shows the logistic regression results, which included all possible influencing factors. This model identified 7 independent predictive factors: age, gender, pain, sleep duration, daily living abilities, self-assessed health status, and cognitive ability.

**Table 2:** Final predictive parameters for depression.

Predictors	Beta Coeff	SE	Odds ratio	95%CI	*p*
(Constant)	2.700	0.349	14.883	7.531–29.625	0.000
Self-rated health (reference group: poor/very poor)					
Very good/good/average	−0.902	0.089	0.406	0.341–0.483	0.000
Age	−0.039	0.004	0.962	0.953–0.970	0.000
Sleep (reference group: <4 h)					
4 h ~ 5.9 h	−0.582	0.132	0.559	0.431–0.723	0.000
6 h ~ 8 h	−0.961	0.133	0.383	0.294–0.496	0.000
>8 h	−1.040	0.136	0.353	0.270–0.461	0.000
Pain status (reference group: non)					
Yes	0.614	0.108	1.848	1.479–2.287	0.000
Activities of daily living (reference group: normal)					
disabled	0.473	0.097	1.604	1.326–1.941	0.000
Cognitive score	0.029	0.006	1.030	1.017–1.043	0.000
Gender (reference group: woman)					
Man	−0.303	0.089	0.739	0.621–0.879	0.000

### 3.3 Construction of the nomogram model

**Figure 2:**
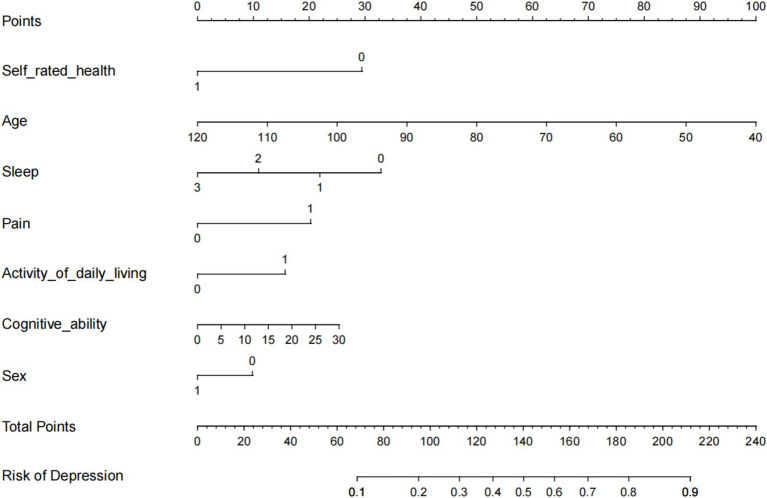
Nomogram to predict the probability of Hearing Impaired Middle-aged and Older Adult Depression.

The seven variables identified as independent predictors of depressive symptoms were included as predictors in the final model, which was developed using R software (version 3.5.2) to create an individualized nomogram (Figure 2). The model shows that the influencing factors for depressive symptoms in middle-aged and older adult patients with hearing loss, in descending order, are: pain status (OR: 1.848), activities of daily living (OR: 1.604), cognitive ability (OR: 1.030), age (OR: 0.962), gender (OR: 0.739), sleep duration (OR: 0.559), and self-rated health status (OR: 0.406). Notably, although the odds ratios for sleep and self-rated health are close to 0.3, numerous studies have confirmed that sleep quality and self-rated health are closely related to the increase in depressive symptoms, and they have also shown statistical significance in our study (20–22). Therefore, we believe that these factors should be included in the final model.

### 3.4 Model validation

**Figure 3:**
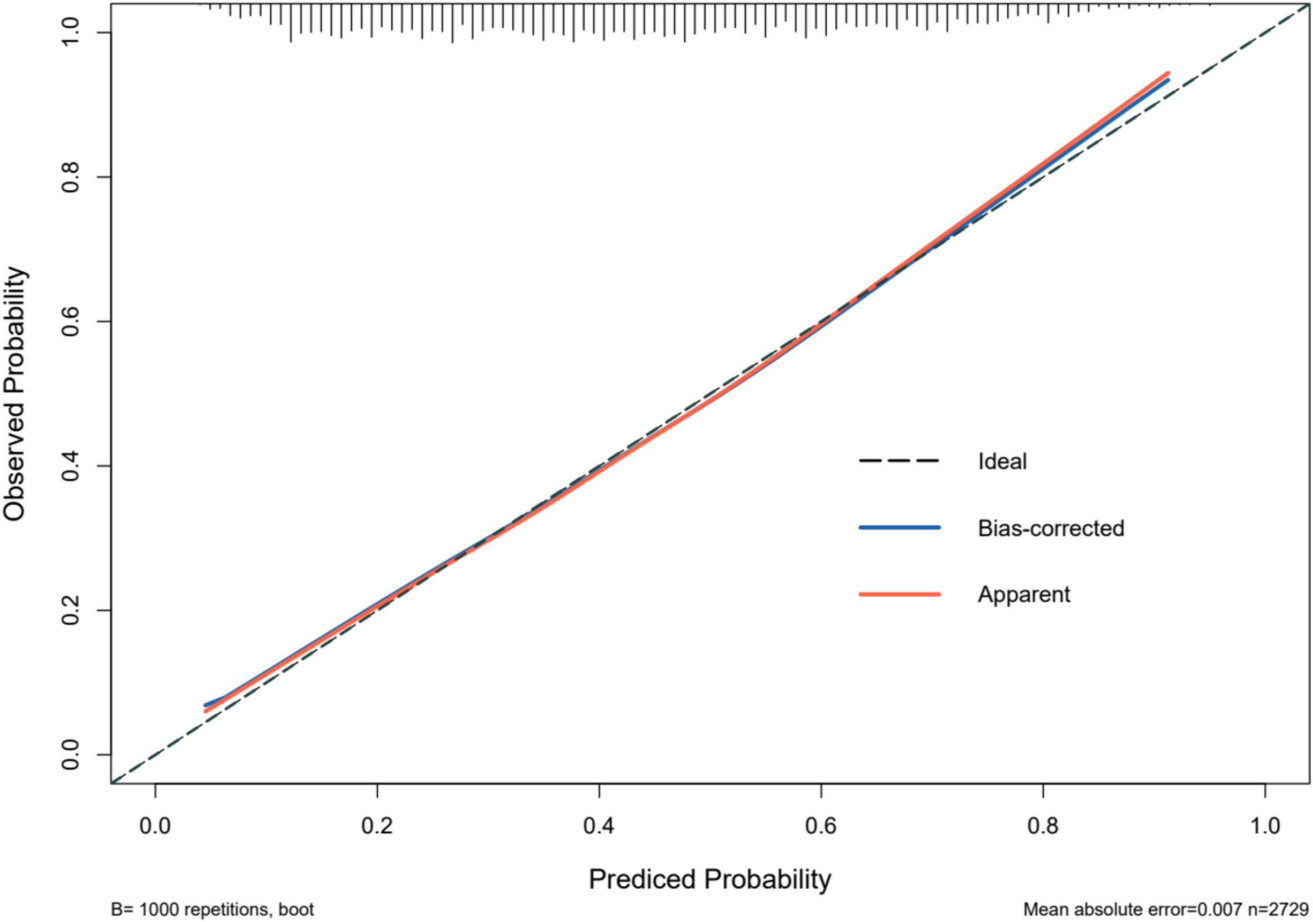
Calibration of the nomogram model for predicting Hearing Impaired Middle-aged and Older Adult Depression risk.

**Figure 4:**
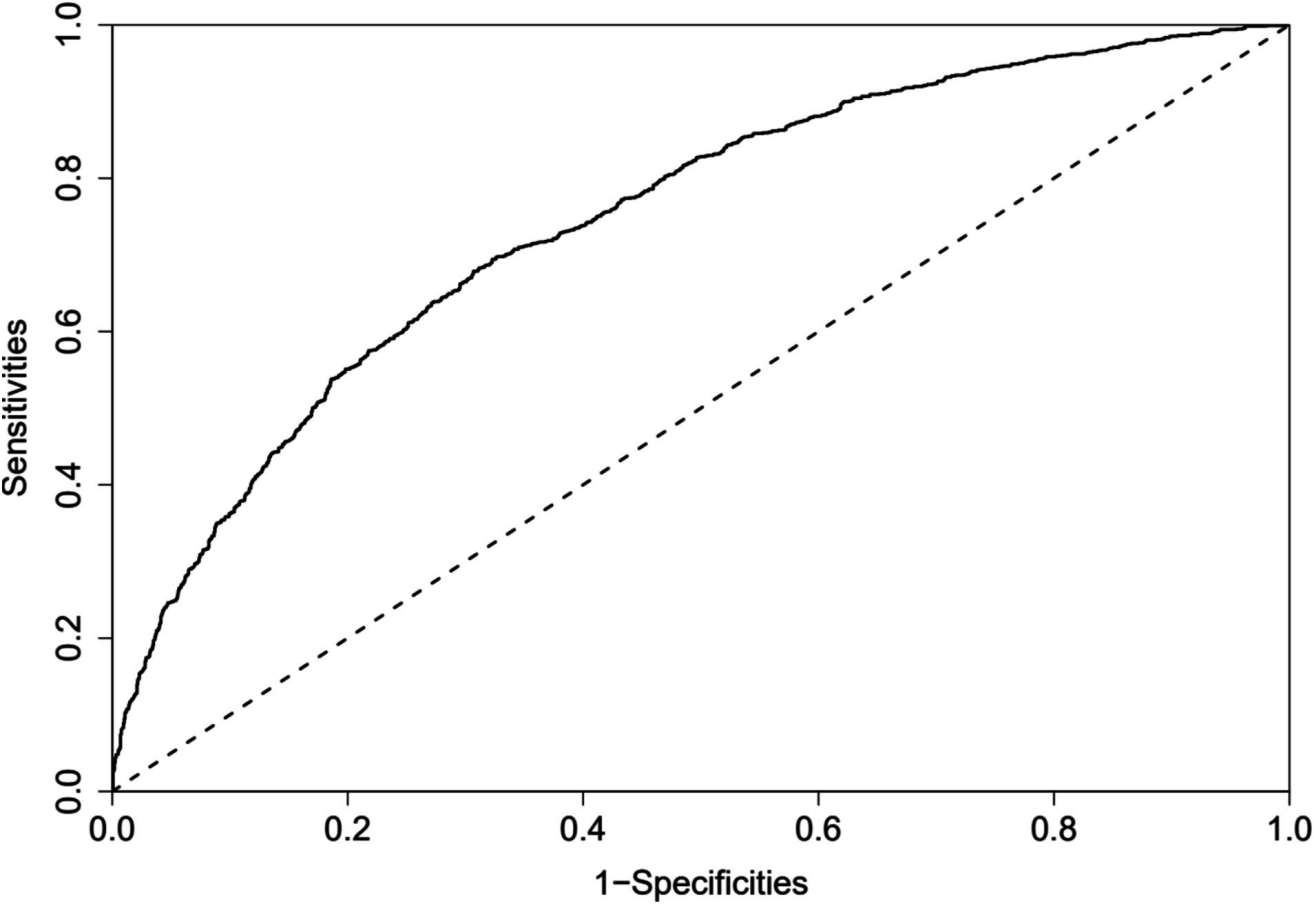
ROC curves for the nomogram model to predict Hearing Impaired Middle-aged and Older Adult Depression risk.

**Figure 5:**
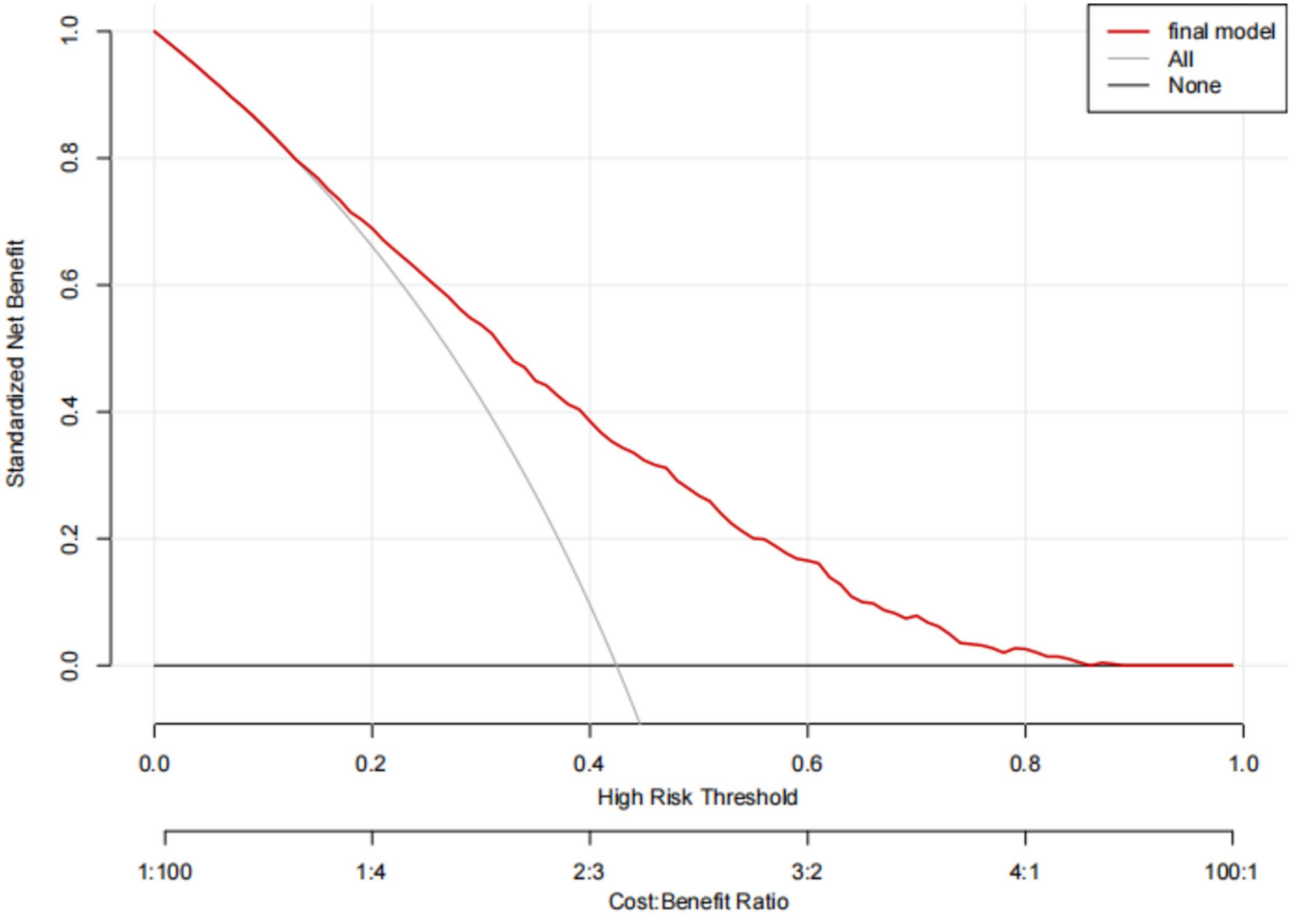
Decision analysis curves for the nomogram model for predicting Hearing Impaired Middle-aged and Older Adult Depression risk.

The predictive model for depressive symptoms in middle-aged and older adult individuals with hearing loss was calibrated using the Hosmer-Lemeshow goodness-of-fit test. The Hosmer-Lemeshow test indicated good calibration of the predictive model (χ^2^ = 3.367, *p* = 0.909). As shown by the calibration curve, there was a high concordance between the predicted probabilities and the observed probabilities (Figure 3). Additionally, the ROC curve, as depicted in Figure 4, showed that the model had an AUC value of 0.741 (95% CI: 0.723–0.759). The Decision Curve Analysis (DCA), shown in Figure 5, illustrates that the curve representing the model’s predictive benefit is consistently above other curves, indicating that the model’s decisions are likely to benefit patients. The predictive accuracy of the nomogram was internally validated using the bootstrap resampling method (1,000 bootstrap resamples), resulting in an average AUC value of 0.683.

## 4 Discussion

The results of this study reveal that the incidence of depressive symptoms among middle-aged and older adult individuals with hearing loss is 42.4%. Depressive symptoms is a widespread mental health issue, with varying prevalence rates across different countries (23). Our findings not only exceed the general depressive symptoms rate of 38.27% observed among the older adult population in China but also far surpass the global average prevalence rate of 10–20% (23, 24). Consequently, there is an urgent need to explore the factors contributing to this condition in such populations and to implement policy interventions based on these factors to alleviate the depressive conditions among the older adult in our country, thereby reducing the social and healthcare burdens caused by this issue.

### 4.1 Factors influencing depressive symptoms in middle-aged and older adult patients with hearing loss

#### 4.1.1 Gender

This study delves into the demographic factors and their relationship with depressive symptoms among the middle-aged and older adult population affected by hearing loss. Our findings indicate that women in this group are more prone to depressive symptoms than men, consistent with most of the relevant research (25–27). The reasons for this gender difference might be twofold: firstly, the pathogenesis and physiological pathology of depressive symptoms may differ inherently between genders. Women are more susceptible to mood disorders during significant hormonal fluctuations such as pregnancy and postpartum than men. Additionally, differences in the hormonal environment between males and females (although hormones in both genders help combat depressive symptoms) may contribute to the varying incidence rates of depressive symptoms (28–30).

Moreover, women tend to exhibit stronger emotional reactions and higher psychological stress when facing health issues, which may be attributed to differences in social roles, family responsibilities, and hormonal factors. Additionally, hearing loss may exacerbate feelings of social isolation and a decline in quality of life for women, thus increasing their risk of depressive symptoms. In contrast, men may be more likely to hide or overlook psychological problems (31). Although they are also affected by hearing loss, their depressive symptoms may be less frequently reported or diagnosed. Therefore, the moderating role of gender in the relationship between hearing loss and depressive symptoms warrants further investigation.

#### 4.1.2 Age

Through multivariate regression analysis, we found that in middle-aged and older adult individuals with hearing loss, the closer their age to 45, the higher their risk of depressive symptoms. This indicates that age is a negative predictor of depressive symptoms in this specific population, consistent with findings by Li et al. (32), Christie-Mizell et al. (33), Solomou et al. (34), but contrary to those by Salk et al. (35). This phenomenon may be related to the characteristics of the population with hearing loss: at age 45, individuals are not fully aged yet, but they bear the burden of raising children, supporting older adult relatives, and fulfilling social responsibilities. At this time, hearing impairments negatively impact one’s work, social interactions, and communication, increasing stressors and thereby the risk of depressive symptoms. However, as age increases and individuals enter retirement, the reduction in stressors provides opportunities for emotional recovery, thus alleviating anxiety and depressive symptoms (36). Therefore, we particularly emphasize the need to focus on the mental health issues of middle-aged women. Although this study identifies age as a negative predictor of depressive symptoms in this population, further research across a broader and full age range is needed to identify and more effectively prevent and intervene in the onset of depressive symptoms.

#### 4.1.3 Sleep

In our study, we explored the connection between lifestyle habits and depressive symptoms, finding that insufficient sleep can increase the risk of depressive symptoms. Firstly, the results indicate that increased sleep duration is negatively correlated with a reduced risk of depressive symptoms, a finding supported by extensive research (21, 37). Furthermore, studies have shown that treating sleep disorders with Cognitive Behavioral Therapy (CBT) and enhancing mood states can effectively prevent the onset of depressive symptoms (38, 39). This suggests that sleep quality is a key risk factor that can be improved through interventions, which is significant for the treatment and prevention of depressive symptoms.

#### 4.1.4 Pain

Consistent with existing research, we consider physical pain to be a significant factor in triggering depressive symptoms, involving complex, multi-layered interactions (40, 41). Research has identified a significant overlap between pain and the neurobiological changes associated with depressive symptoms, which is crucial for understanding the development and progression of depressive symptoms (42). Persistent pain can cause the body to remain in a state of stress over the long term, increasing levels of stress hormones and promoting the development of depressive moods; effectively managing pain can significantly reduce this physiological stress and, in turn, decrease the risk of depressive symptoms (43).

#### 4.1.5 Daily living abilities

Regarding limitations in daily living abilities, our findings indicate that this is a positive predictor of depressive symptoms. Extensive research has shown that impairments in the function of daily activities are highly correlated with depressive symptoms, with a bidirectional influence between the two (44, 45). Particularly for middle-aged and older adult individuals, hearing loss not only increases the risk of functional impairments in daily activities but also the likelihood of developing depressive symptoms. Although existing studies have indicated independent associations between hearing impairments, depressive symptoms, and functional disabilities, research on their specific relationships is still scarce and lacks a consensus view (46).

#### 4.1.6 Self-assessed health status

In our study, we explored the relationship between psychological and mental health factors and depressive symptoms, finding that individuals who rated their health as poor were more susceptible to depressive symptoms. This finding aligns with numerous studies in public health and psychology, suggesting that poor self-assessed health may not only reflect actual health problems, but these health issues could themselves trigger or exacerbate depressive symptoms. Conversely, individuals who rate their health as good are likely to enjoy better physical health and a higher quality of life, factors that can enhance psychological satisfaction and well-being, thereby helping to reduce the risk of depressive symptoms (47).

#### 4.1.7 Cognitive abilities

Our research also points out that cognitive abilities are a positive predictor of depressive symptoms, particularly evident in the middle-aged and older adult population. Individuals with higher cognitive abilities may have a keener perception of their health status, including the impact of hearing loss (48). Compared to the general older adult population, they may be more sensitive to the limitations hearing loss imposes on daily life and social activities, leading to more intense feelings of frustration and depressive moods. These findings highlight the complex interplay between psychological and physical health and underscore the importance of enhancing awareness and management of health conditions, especially in the prevention and mitigation of depressive symptoms.

### 4.2 Implications for interventions

Based on the findings of this study, it is recommended to adopt a comprehensive approach for the prevention and treatment of depressive symptoms in middle-aged and older adult patients with hearing loss, combining hearing interventions (such as hearing aids) with psychological therapies (such as cognitive behavioral therapy). This approach may be particularly effective for high-risk groups, such as women, middle-aged individuals around 45 years old, and patients with higher cognitive abilities. A multidimensional intervention strategy, including hearing aids, sleep improvement, and pain management, could provide better outcomes for these individuals.

### 4.3 Construction of a nomogram model

This study summarized the factors associated with depressive symptoms among middle-aged and older adult individuals with hearing loss and used seven risk factors as predictors to construct a nomogram model. After validating the model, we found that its calibration results align closely with the ideal curve. The nomogram model’s ROC curve area under the curve (AUC) is high, indicating good discriminative ability. The Hosmer-Lemeshow test shows that the predictive data of the model is nearly identical to the actual data, indicating high calibration or predictive accuracy. The nomogram model illustrates the relationships between depressive symptoms and seven variables: age (OR: 0.962), sex (OR: 0.739), daily living abilities (OR: 1.604), self-assessed health (OR: 0.406), cognitive abilities (OR: 1.030), sleep (OR: 0.559), and pain (OR: 1.848). It is readable and easy to evaluate. This nomogram model can assist healthcare providers in intuitively and conveniently identifying the risk factors for depressive symptoms and assessing the risk of depressive symptoms in this population, thereby helping to guide targeted intervention measures to reduce the incidence of depressive symptoms.

regarding the practical application of the model, we believe that the nomogram can serve as an effective tool for physicians in assessing the risk of depressive symptoms in middle-aged and older adult patients with hearing loss. To achieve this, healthcare providers may need to undergo basic training to ensure they can proficiently use the nomogram for individualized risk assessment. Furthermore, this model can be integrated into existing electronic health record systems, streamlining the process and improving clinical efficiency. During future implementation, we also recommend conducting relevant training and educational programs to help healthcare professionals fully understand and utilize the tool effectively.

## 5 Limitations

This study has several limitations. First, it cannot determine the severity or type of depressive symptoms, as the study only used the CES-D10 scale to assess the presence of depressive symptoms. Future research needs to refine the types of depressive symptoms and explore their respective influencing factors. Second, the measurement of hearing loss in this study relied on subjective self-assessment of hearing ability, which may introduce bias in the selection of the population. Future studies could use audiometric testing equipment to more accurately assess hearing loss. Third, this is a cross-sectional study, thus it cannot establish causality between the risk factors for depressive symptoms and hearing loss in the older adult. Therefore, future research should be longitudinal to achieve this goal.

## 6 Conclusion

In conclusion, This study constructed a predictive model for depressive symptoms among older adult patients with hearing loss, which includes seven independent predictive factors: age, gender, daily living capabilities, pain, cognitive function, sleep, and self-assessed health. Upon evaluation, this model demonstrated good discriminatory and predictive capabilities, and can serve as a reference for community healthcare and clinical prediction of patients at high risk for depressive symptoms. In addition, our study emphasizes that to more effectively prevent and treat depressive symptoms, we should not only focus on individual health conditions or lifestyle behaviors but should implement comprehensive, multimodal intervention measures from multiple angles to achieve better synergistic effects. We anticipate that future research will clarify the specific connections and interaction pathways between these factors, thus providing more effective intervention strategies to reduce the incidence of depressive symptoms.

## Data Availability

The raw data supporting the conclusions of this article will be made available by the authors, without undue reservation.
